# Predictor parameters for poor prognosis in patients with sudden sensorineural hearing loss: fibrinogen to albumin ratio vs C-reactive protein to albumin ratio

**DOI:** 10.1016/j.bjorl.2020.06.010

**Published:** 2020-07-27

**Authors:** Serkan Cayir, Serkan Kayabasi, Omer Hizli

**Affiliations:** a Aksaray University, Aksaray Education and Research Hospital, Department of ENT, Aksaray, Turkey; b Aksaray University, Faculty of Medicine, Department of ENT, Aksaray, Turkey; c Giresun University, Prof Dr. A. Ilhan Ozdemir Education and Research Hospital, Department of ENT, Giresun, Turkey

**Keywords:** Sudden sensorineural hearing loss, Fibrinogen, C-reactive protein, Albumin

## Abstract

**Introduction:**

Prognosis of sudden sensorineural hearing loss may be predicted using several parameters of laboratory blood analysis.

**Objective:**

To identify and investigate the most significant indicator parameters related to the poor prognosis of sudden sensorineural hearing loss.

**Methods:**

Eighty-eight patients were included, and three groups were constituted: non-recovery group with14 patients, recovery group with 33 patients and control group with 41 individuals. We compared fibrinogen-to-albumin ratio, C-reactive protein-to-albumin ratio, neutrophil-to-lymphocyte ratio, platelet-to-lymphocyte ratio, white blood cell and hemoglobin of the groups. Then, we investigated the most significant indicator parameters related to the poor prognosis of sudden hearing loss.

**Results:**

The mean hemoglobin, mean platelet-lymphocyte ratio and median white blood cell values did not significantly differ among three groups (*p* = 0.36, *p* = 0.86 and *p* = 0.79, respectively). A significant difference of median fibrinogen-albumin ratio, C-reactive protein-albumin ratio, neutrophil-to-lymphocyte ratio was evident among three groups (*p* < 0.001, *p* = 0.003 and *p* = 0.002, respectively). Median fibrinogen-albumin ratio, C-reactive protein-albumin ratio and neutrophil-to-lymphocyte ratio values were significantly greater in the non-recovery group, compared with the controls (*p* < 0.001, *p* = 0.003 and *p* = 0.005, respectively). Median fibrinogen-to-albumin ratio, C-reactive protein-to-albumin ratio and neutrophil-to-lymphocyte ratio were significantly greater in the recovery group, compared with the controls (*p* < 0.001, *p* = 0.013 and *p* = 0.005, respectively). Moreover, the median fibrinogen-albumin ratio was significantly greater in the non-recovery group compared with the recovery group (*p* = 0.017). However, no statistically significant difference of median C-reactive protein-albumin ratio, neutrophil-to-lymphocyte was evident between the non-recovery and recovery groups (*p* = 0.15).

**Conclusion:**

Increased levels of fibrinogen-albumin ratio may be predictive for poor prognosis in patients with sudden sensorineural hearing loss.

## Introduction

Sudden sensorineural hearing loss (SSNHL) is described as sudden hearing impairment that occurs as an increase of at least 30 dB in hearing thresholds at three bone conduction frequencies within 3 days.^1^ The yearly incidence of SSNHL is reported as 5–20/100,000 and it is estimated that recurrence usually occurs in 30%–40% of these patients.^2^ A wide variety of SSNHL causes have been postulated, including bacterial or viral infections, microvascular circulatory disorders, metabolic conditions, labyrinthine membrane rupture, neoplastic events, and autoimmune disorders. Each of these etiological factors has been suggested based on various laboratory and postmortem findings, but none could fully clarify the pathogenesis of SSNHL. Therefore, the main etiologic factor of SSNHL is still a matter of debate.

Chronic inflammation plays an essential role in recent studies focused on the mechanism of formation of SSNHL. Chronic inflammation increases the risk of ischemia by causing atherogenesis and microvascular injury.^3^ The blood supply of the cochlea is basically provided by a single terminal artery, the labyrinthine artery. In addition, cochlear cells have high oxygen consumption and are highly susceptible to hypoxia. Therefore, the sac cells in the cochlea are prone to be affected by the microcirculation changes. Thus, factors such as chronic inflammation that can cause microcirculation disorder in the inner ear may be closely related to the pathophysiology of SSNHL.^4^

Plasma fibrinogen, an acute phase reactant, is the most important coagulation protein increasing in inflammatory situations.^5^ Serum levels of albumin, a negative acute phase protein, tend to drop in cases of acute inflammation, but are mainly reduced in malnutrition and chronic inflammatory processes. In several studies, the Fibrinogen-Albumin Ratio (FAR) was determined to be a predictive marker for the severity of various types of diseases.^6^, ^7^

In recent studies, 8, 9 neutrophil-to-lymphocyte ratio (NLR), platelet-lymphocyte ratio (PLR) and C-reactive protein (CRP)-albumin ratio (CAR), showing chronic inflammation, were reported to be related to the prognosis of the patients with SSNHL. To the best of our knowledge, none of them have examined the relationship between FAR and SSNHL.

The purpose of this cross-sectional and retrospective analysis was to clarify the relationship between FAR and poor prognosis in patients with SSNHL. In addition, we investigated the association of SSNHL with many parameters of blood test like FAR, CAR, NLR, PLR, White Blood Cell (WBC) and hemoglobin. Moreover, we investigated the most significant predictor parameter of poor prognosis of SSNHL.

## Methods

### Participants and design

This cross-sectional and retrospective study was conducted in conformity with the Helsinki Declaration. Approval of the local ethical committee was obtained from Aksaray University (2019/10-06). After searching the medical archives, we identified the patients with SSNHL. The patients with any otologic surgery history, a nutritional and/or inflammatory disease that might alter the level of blood parameters, another otologic disease that might affect hearing, and the patients admitted 5 days (or later) after the initiation of SSNHL were excluded.

Study groups were composed of the patients with SSNHL, and the control group was composed of healthy individuals. To all patients with SSNHL, a treatment of corticosteroid (prednisone with an initial dose of 1 mg/kg/day) was applied with a gradual dose reduction maintained for at least 2 weeks. The patients were categorized according to the Siegel criteria^10^ and 2 groups were constituted: The recovery group (Type 1, 2, 3) and the non-recovery group (Type 4). During the treatment, hearing levels of the patients were followed-up by audiometric evaluations performed at the first day of the treatment and 3 months after the treatment (AC40, Interacoustic, Denmark).

### Laboratory evaluation

Laboratory blood tests of all patients were performed on the first day of admission, before initiating any treatment. Complete blood count measurements were performed using an automated blood cell counter (Mindray BC-6000, Shenzhen, China). Serum albumin levels were detected using automatic photometry commercial kits (Abbott C8000i, Abbott Park, IL). Serum CRP levels were detected using the nephelometric method (AU5800 System; Beckman Coulter Inc, Brea, CA) and serum fibrinogen levels were detected by Clauss method using a BCS Analyzer (Multifibren *U*; Siemens Healthcare, Erlangen, Germany). We retrospectively recorded the pre-treatment fibrinogen, CRP, WBC, hemoglobin, neutrophil, lymphocyte, platelet, and albumin levels of the patients and calculated FAR, CAR, NLR and PLR of the groups.

First, the comparisons of FAR, CAR, NLR, PLR, WBC and hemoglobin were performed among the groups. Then, the most significant indicator parameters of poor prognosis (non-recovery) in patients with SSNHL were statistically investigated.

### Statistical analysis

Results were presented as median (min–max) for abnormally distributed data while mean + standard deviation for normally distributed data. Data were investigated using Kolmogorov-Smirnov test to determine the distribution pattern. The data of hemoglobin and PLR had a normal distribution (*p* > 0.05), thus, the comparisons of mean hemoglobin and PLR were performed using the one-way analysis of variance test (ANOVA). The data of FAR, CAR, NLR, WBC did not have a normal distribution (*p* < 0.05), thus, Kruskal–Wallis test was used for the comparison of median FAR, CAR, NLR and WBC of the groups. The post hoc test used for advanced comparisons of median FAR, CAR and NLR values was Mann–Whitney *U* test. To detect the most significant parameter related to the poor prognosis of SHL, and to determine a cut-off value, Receiving Operator Characteristics (ROC) curve analysis test was used. All statistical analysis was performed using SPSS 23.0 software for Windows (SPSS Inc., Chicago, IL). A *p*-value < 0.05 was considered to represent a statistical significance. Additionally, for post-hoc comparison tests, Bonferroni correction rule (three groups = triple combination) was applied and a *p*-value < 0.017 (0.05/3) was considered to represent a statistical significance.

## Results

Forty-seven patients with SSNHL and 41 controls were included in the study. The non-recovery group was composed of 14 patients (6 males/8 females with a mean age of 43 ± 10 years), the recovery group was composed of 33 patients (18 males/15 females, with a mean age of 42 ± 9 years) and the control group was composed of 41 individuals (19 males/22 females, with a mean age of 43 ± 9 years). The groups were age and gender-matched (*p* = 0.8, *p* = 0.69). Our recovery rate was 70.2%.

The mean hemoglobin and PLR values of the groups were shown in Table 1. One-way ANOVA revealed that the mean hemoglobin and PLR were not significantly different among the groups (*p* = 0.36 and *p* = 0.86, respectively).

**Table 1:** The mean Hgb and PLR values of the groups.

	Hgb (g/dL)	PLR
Control group	13.96 ± 0.27	142.6 ± 31.5
Recovery group	14.12 ± 0.65	138.5 ± 31.4
Non-recovery group	14.03 ± 0.49	141.5 ± 34.7
*p*-Value	0.36	0.86

Hgb, hemoglobin; PLR, platelet to lymphocyte ratio.

The median FAR, CAR, NLR and WBC of the groups were shown in Table 2. According to the Kruskal–Wallis analysis, median WBC was not significantly different among the groups (*p* = 0.79). However, a statistically significant difference of median FAR, CAR and NLR was evident among three groups (*p* < 0.001, *p* = 0.003 and *p* = 0.002, respectively).

**Table 2:** The median FAR, CAR, NLR and WBC values of the groups.

	FAR	CAR	NLR	WBC (K/μL)
Control	51.56 (34.12–138.6)	0.5 (0.21–1.51)	1.77 (1.3–3.1)	8.14 (6.02–9.64)
Recovery group	69.96 (43.03–134.84)	0.73 (0.19–1.64)	2 (1.63–3.5)	8.56 (7.13–9.24)
Non-recovery group	85.22 (64.65–139.02)	0.89 (0.25–2.19)	2.61 (1.5–3.3)	8.28 (7.14–9.31)
*p*-Value	<0.001	0.003	0.02	0.79

FAR, fibrinogen to albumin ratio; CAR, C-reactive protein to albumin ratio; NLR, neutrophil to lymphocyte ratio; WBC, white blood cell.

Advanced comparisons revealed that median FAR, CAR and NLR values were significantly greater in the non-recovery group, compared with the control group (*p* < 0.001, *p* = 0.003 and *p* = 0.005, respectively). Additionally, median FAR, CAR and NLR values were significantly greater in recovery group, compared with the control group (*p* < 0.001, *p* = 0.013 and *p* = 0.005, respectively). Moreover, the median FAR was significantly greater in the non-recovery group compared with the recovery group (*p* = 0.017). However, no statistically significant difference of median CAR and NLR was evident between recovery and non-recovery groups (*p* = 0.15).

**Figure 1:**
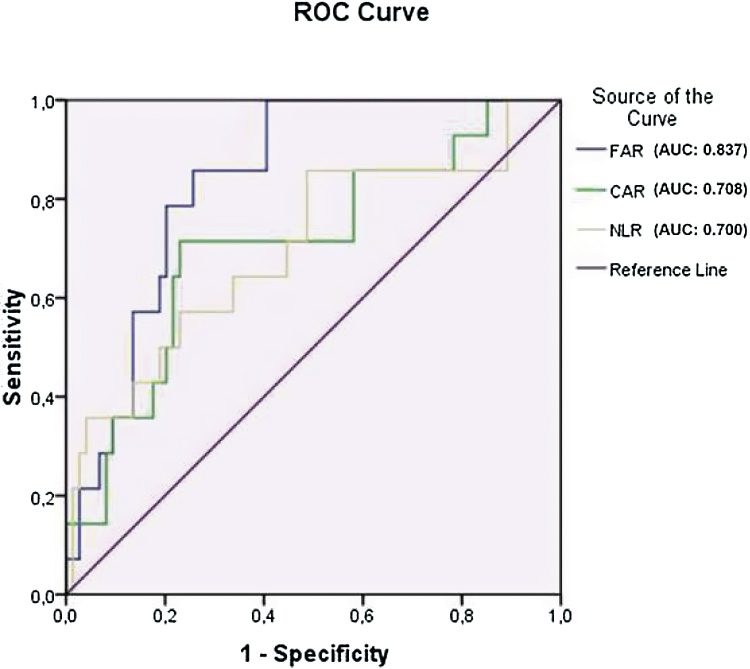
ROC curve analysis graph of non-recovery SSNHL.

Fig. 1 is the graph of the ROC curve analysis for unrecovered SSNHL. The area under ROC curve of FAR (0.837 [95% CI: 0.75–0.93, *p* < 0.001]) was greater compared both with CAR (0.708 [95% CI: 0.56–0.86, *p* = 0.014]) and NLR (0.700 [95% CI: 0.54–0.86, *p* = 0.014]). The cut-off value of FAR indicating poor prognosis in SSNHL was detected as 76.08 (sensitivity: 79% and specificity: 80%).

## Discussion

Increased levels of inflammatory markers like FAR may have a clinical implication as being related to the severity and prognosis of SSNHL. In this study, the FAR levels of the patients with non-recovery SSNHL were significantly greater compared with the control patients. Our results suggested that FAR might be the predictor parameter of non-recovery SSNHL.

SSNHL, often described as sudden deafness, occurs as an unexplained, rapid hearing loss, and usually occurs in one ear, at once or within a few days. It is considered as a medical emergency in otolaryngology practice. There exist various treatment modalities used to recover SSNHL, and various approaches have been proposed, such as steroids, antivirals, vasodilators, low-salt diets, and diuretics. However, the rate of spontaneous recovery without treatment was reported between 30% and 60%, and improvement is usually seen within the first 2 weeks.^11^ Although the most effective method of treating SSNHL was systemic steroid therapy because of its anti-inflammatory effect, previous studies reported that approximately 30%–40% of patients did not respond to oral or intravenous therapy.^11^ We found a recovery rate of 70.2% in our patients, consistent with the prior literature.

The etiopathogenesis of SSNHL is not completely clear, it is often described as idiopathic, and the exact cause is still not fully determined. Many hypotheses in the past have attempted to explain the cause of SSNHL; eventually, the inflammation, viral infection and hypoxia have been the most emphasized ones.^12^ Prognostic factors in SSNHL have long been investigated and several parameters like the level of hearing impairment, the presence of vertigo, the type of audiogram, status of the contralateral ear, and the time of onset of treatment were considered to be related to poor prognosis of SSNHL.^13^

In recent studies, the prognostic value of various hematologic parameters like CAR, NLR, PLR and RDW on the patients with SSNHL was investigated, and the relationship between these markers and SSNHL prognosis was determined.^8^, ^9^ Consistent with these studies, we found that NLR and CAR were significantly higher in the recovery group than in the control group. However, WBC, hemoglobin, and PLR were not significantly different among the groups. In the recent report by Ocal et al.,^8^ CAR was found as being increased in the patients with SSNHL compared to the controls, but not associated with the response to the treatment. CAR might show the inflammatory base of SSNHL, but the clinical implication of CAR would have appeared if it had been greater in patients without recovery compared to the patients with recovery. Unlike this report,^8^ we compared the FAR levels of the patients with and without recovery to the control group in our study. FAR levels were significantly greater in patients without recovery, compared both to the patients with recovery and the controls. Additionally, FAR had a significantly greater area under curve value in our ROC curve analysis. Hence, although CAR was found as associated with SSNHL, FAR might be a more significant predictive parameter associated with the poor prognosis in SSNHL.

Fibrinogen, a plasma glycoprotein synthesized hepatically, is the most abundant coagulation factor found in the human body.^14^ Fibrinogen levels usually increase as an acute phase reactant in advanced cardiogenic disorders and high fibrinogen levels were reported in patients requiring mechanical circulatory support.^15^ Increased fibrinogen levels are related to a high level of fibrin formation, thrombus fibrin content, fibrin network stability, and platelet aggregation.^16^ Acute phase proteins are defined as proteins that increase or decrease by at least 25 percent during inflammatory states, these are referred to as either positive or negative Acute Phase Reactants (APR), respectively. Some cytokines like TNF-α, IL-1β, IL-6 and interferon-γ suppress the synthesis of albumin, consequently, serum albumin levels decrease in inflammation, thus albumin is a negative APR.^17^ An elevated FAR has been defined as a marker of inflammation and disease severity in patients with ST elevation myocardial infarction, chronic venous insufficiency, ischemic retinal vein occlusion, and in patients with various inflammatory disorders.^7^, ^18^, ^19^ However, to the best of our knowledge, FAR levels and the prognostic value of FAR in patients with SSNHL have not been investigated. In our research, FAR values were significantly higher in patients with SSNHL compared to the control group, indicating the presence of inflammation. In addition, FAR values were significantly higher in the non-recovery group compared both to the recovery group and the control group. Thus, increased levels of FAR may help physicians as a convenient and dependable indicator to predict the prognosis of SSNHL. Additionally, the cut-off value of FAR for poor prognosis in SSNHL was detected as 76.08, with a sensitivity of 79% and specificity of 80% in our ROC test. Therefore, patients with SSNHL with a FAR value greater than 76.08 might be considered candidates for non-recovery. Results of our study revealed that CAR and NLR levels were increased in patients with SSNHL as well as FAR. However, the CAR and NLR were not significantly related to recovery. Furthermore, elevated CAR and NLR might also be a poor prognosis indicator in patients with SSNHL, but FAR had a greater area under the ROC curve. These results suggest that elevated FAR levels in patients with SSNHL might be more valuable than other parameters to predict the prognosis.

As far as we know, this is the first study investigating the predictive value of FAR in patients with SSNHL and giving a cut-off value indicating poor prognosis. Given the inflammatory and microcirculatory fundamentals of the causative mechanism of SSNHL, assessment of FAR values before initiating a treatment might be logical, in order to predict the poor prognosis and more clearly inform the patients. However, the study had certain limitations: the sample size was relatively small, and data was compiled retrospectively. Thus, further-multicenter prospective studies are needed to confirm our results.

## Conclusion

In conclusion, increased FAR values in patients with SSNHL might be considered as a one of the novel potential parameters to predict the prognosis. The cut-off value of FAR for poor prognosis in SSNHL was 76.08, with a sensitivity of 79% and specificity of 80%. Hence, patients with SSNHL with a FAR value greater than 76.08 might be considered as related to poor prognosis.

## Ethical approval

All procedures performed in this study were in accordance with the ethical standards of local ethical committee of Aksaray University (IRB Number: 2019/10-06).

## Financial support

The authors declared that this study has received no financial support.

## Conflicts of interest

The authors declare no conflicts of interest.
